# Benchtop NMR Coupled with Chemometrics: A Workflow for Unveiling Hidden Drug Ingredients in Honey-Based Supplements

**DOI:** 10.3390/molecules29092086

**Published:** 2024-05-01

**Authors:** Camille Pujol, Saïda Danoun, Ghislaine Biasini, Emmanuel Retailleau, Jessica Masson, Stéphane Balayssac, Véronique Gilard

**Affiliations:** 1 Laboratoire Softmat, Université de Toulouse, CNRS UMR 5623, Université Toulouse III—Paul Sabatier, 31062 Toulouse, France; camille.pujol1@univ-tlse3.fr; 2 Laboratoire SPCMIB, Université de Toulouse, CNRS UMR 5068, Université Toulouse III—Paul Sabatier, 31062 Toulouse, France; saadia.danoun@univ-tlse3.fr; 3 Département de Chimie, Université de Toulouse, Université Toulouse III—Paul Sabatier, 31062 Toulouse, France; ghislaine.biasini@univ-tlse3.fr (G.B.); emmanuel.retailleau@univ-tlse3.fr (E.R.); 4 SCL, Laboratoire d’Île-de-France, 25 Avenue de la République, 91300 Massy, France; jessica.masson@scl.finances.gouv.fr

**Keywords:** low-field NMR, compact NMR, benchtop NMR, honey, adulteration, sildenafil, tadalafil, chemometrics

## Abstract

Recently, benchtop nuclear magnetic resonance (NMR) spectrometers utilizing permanent magnets have emerged as versatile tools with applications across various fields, including food and pharmaceuticals. Their efficacy is further enhanced when coupled with chemometric methods. This study presents an innovative approach to leveraging a compact benchtop NMR spectrometer coupled with chemometrics for screening honey-based food supplements adulterated with active pharmaceutical ingredients. Initially, fifty samples seized by French customs were analyzed using a 60 MHz benchtop spectrometer. The investigation unveiled the presence of tadalafil in 37 samples, sildenafil in 5 samples, and a combination of flibanserin with tadalafil in 1 sample. After conducting comprehensive qualitative and quantitative characterization of the samples, we propose a chemometric workflow to provide an efficient screening of honey samples using the NMR dataset. This pipeline, utilizing partial least squares discriminant analysis (PLS-DA) models, enables the classification of samples as either adulterated or non-adulterated, as well as the identification of the presence of tadalafil or sildenafil. Additionally, PLS regression models are employed to predict the quantitative content of these adulterants. Through blind analysis, this workflow allows for the detection and quantification of adulterants in these honey supplements.

## 1. Introduction 

Over the past twenty years, cases of adulteration of aphrodisiac dietary supplements have been regularly reported [1]. Active pharmaceutical ingredients of phosphodiesterase-5 inhibitor drugs designed for erectile dysfunction, like sildenafil (Viagra™) or tadalafil (Cialis™), were commonly used as adulterants. The first case of adulteration by sildenafil was reported in 2002 in a dietary supplement capsule claimed to be an extract of animal organs and traditional Chinese herbs [2]. Then, until recently, numerous studies reported the use of adulteration of food supplements commercialized as natural preparations with approved phosphodiesterase-5 (PDE-5) inhibitor active pharmaceutical ingredients or their chemical analogs [3,4,5,6,7,8]. 

Several analytical methods were implemented for the identification and characterization of PDE-5 inhibitor adulterants in food supplements [4,9,10]. The most powerful analytical methods for identification and structure identification were liquid chromatography–tandem mass spectrometry (LC-MS/MS) in association with high-field (HF) nuclear magnetic resonance (NMR). Quantification of these adulterants is less obvious but can be easily performed with high-performance liquid chromatography with UV detection (HPLC-UV) [11], LC-MS/MS [7] or gas chromatography–tandem mass spectrometry (GC-MS/MS) [12] when reference compounds of adulterants are available. For new analogs for which no analytical standard is available, HF NMR is more appropriate insofar as the signal area is proportional to the number of nuclei; the technique does not require a specific standard [3]. Low-field (LF) or benchtop NMR spectroscopy stands as a cryogen-free NMR technique, showcasing promising outcomes across diverse applications [13,14], particularly in the field of quality control analysis in the pharmaceutical or food industries [15,16]. In recent years, the utilization of benchtop NMR spectroscopy in conjunction with advanced chemometric methods has gained traction as an area of scientific inquiry [17]. For instance, the integration of benchtop NMR with chemometric approaches has proven successful in detecting adulteration in edible oils [18] or food supplements [19].

Recently, new aphrodisiac preparations of honey were commercialized. A case of patients presenting acute cardiovascular disorder caused by such a product illegally containing a PDE-5 inhibitor was reported in 2022 [20] Meanwhile, in 2021, a case of central serous chorioretinopathy was described [21]. The U.S. Food and Drug Administration (FDA) reported in its “Health fraud product” database 23 cases of honey adulteration with sildenafil or tadalafil between 2017 and 2023 [22]. Due to the potential for serious health issues for these recreational food products, control and regulation of these types of honey should be implemented.

The aim of this study was to propose an innovative analytical workflow using a compact benchtop NMR spectrometer combined with chemometric tools for the screening of adulterated honey and the quantification of adulterants. We conducted our analysis on fifty honey samples seized by French customs using a benchtop 60 MHz NMR spectrometer. 

## 2. Results and Discussion

### 2.1. Setting up the Experimental Conditions

#### 2.1.1. Sample Preparation Optimization

Preliminary extraction tests were performed as described in the experimental part on samples **1**, **5** and **6** containing tadalafil and on samples **2** and **4** containing sildenafil. Our goal was to both optimize the extraction of a possible adulterant and to minimize that of interfering matrix signals, i.e., sugars. After different tests, the solvent selected was the mixture methanol-d4:CDCl_3_ (60:40, *v*/*v*). The use of this solvent involves a biphasic extraction since the honey matrix is essentially aqueous. The upper phase contains compounds of interest as well as remaining sugars in a mixture of CDCl_3_ and methanol-d4. 

For the quantitative part of this study, extraction must be total. To implement this methodology of extraction, three successive extractions were performed. We demonstrated that only two successive extractions were sufficient. Indeed, for sildenafil, extraction recoveries were 91% in the first extraction and 9% in the second one, whereas 92% and 8% were obtained for tadalafil. No adulterant was detected in the third extraction in our experimental conditions. For all the analyses described below, two successive extractions are carried out and pooled as described in Section 3.2.2. 

#### 2.1.2. NMR Experiments Optimization

**Figure 1:**
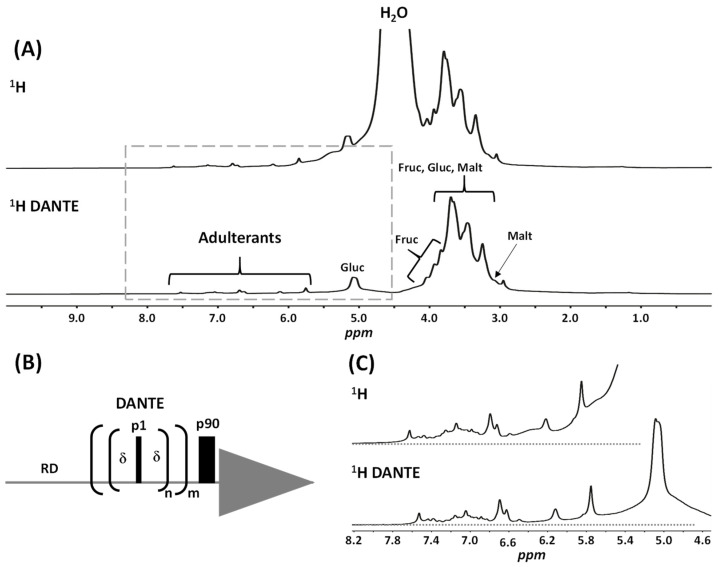
NMR optimization. (**A**) Comparison of a classical (relaxation delay–pulse–acquisition) and DANTE sequence for ^1^H NMR 60 MHz spectra recording of sample **11** after extraction in methanol-d4:CDCl_3_ (60:40, *v*/*v*). (**B**) DANTE sequence with RD: relaxation delay, δ: DANTE delay, p1: low amplitude pulse, p90: 90° pulse, m and n: number of repetitions. (**C**) Zoom on the framed area on the (**A**) spectra for better visualization of the drift in the baseline for both sequences. Spectra shown are raw spectra before applying baseline correction. Gluc: glucose, Fruc: fructose, Malt: maltose.

Benchtop ^1^H NMR analysis of such a complex matrix is quite challenging. Figure 1A represents the whole spectrum of sample **11** using the simplest acquisition pulse sequence (relaxation delay–pulse–acquisition). Obviously, the most intense signal is water, with an intensity around 9 times higher than signals of sugars and 150 times higher than tadalafil. Therefore, it overshadows a large part of the spectrum. To eliminate this water signal, different approaches can be applied, depending on the NMR spectrometer. In the equipment utilized, low gradients limit the use of sequences for water suppression, such as the WATERGATE sequence. Therefore, we have opted for a DANTE sequence, which is shown in Figure 1B. The resulting ^1^H spectrum with water suppression reveals the presence of intense signals of sugars. The other benefit of suppressing the water signal is that it improves the baseline, making it easier to detect any adulterants, as can be observed in Figure 1C. Baseline drift is attenuated by the DANTE sequence, thereby facilitating the integration of signals.

### 2.2. Qualitative Analysis

#### 2.2.1. Assignment of ^1^ H NMR Signals 

In Figure 1A, a typical spectrum with a focus on the sugar signals is presented. It is noteworthy that the objective of this article is not to discuss the composition of honey used as the base matrix of these aphrodisiac supplements. Nevertheless, key markers of honey sugars such as glucose, fructose and maltose are detected. The observed NMR profile aligns with findings recently reported in a study emphasizing honey quality control using benchtop NMR [23]. In the present study, slight variations in sugar composition were observed between samples, but overall, differences were minor and not of specific interest within the scope of this work.

**Figure 2:**
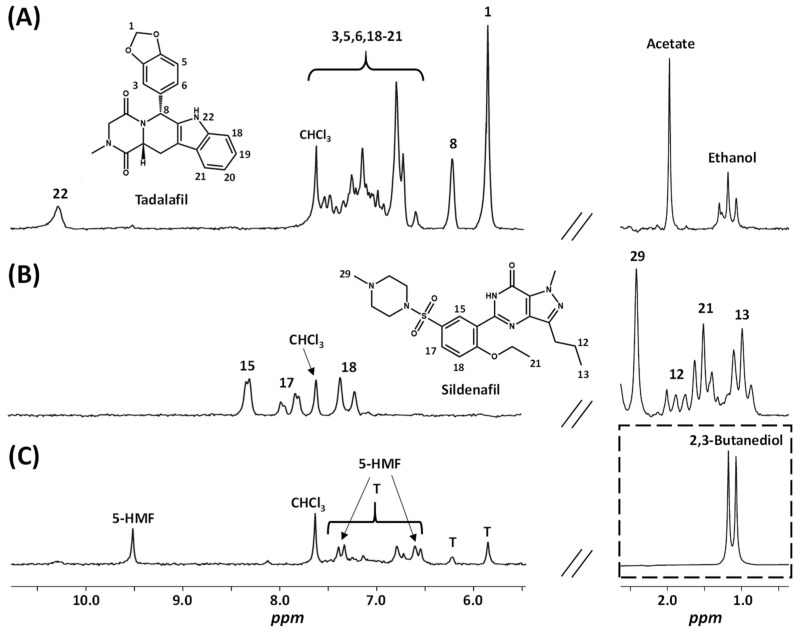
^1^H NMR 60 MHz spectra of honey samples after extraction in methanol-d4:CDCl_3_ (60:40, *v*/*v*) with zooms on the regions 10.7–5.5 ppm and 2.6–0.4 ppm. (**A**) Sample **15**; (**B**) Sample **2**; and (**C**) Sample **37** with T: tadalafil. The intensity of the spectral region delineated by the dotted lines is decreased by a factor of 8.

The NMR analysis of the fifty samples primarily reveals two adulterants. Two spectra representative of such adulteration are reported in Figure 2. Despite some signals being masked by the intense signals of sugars, typical signals are detected. For tadalafil, as evidenced in the spectrum of sample **15** Figure 2A, two characteristic singlets of protons H1 and H8 are detected at 5.85 and 6.22 ppm, respectively, along with a broad NH (H22) signal at 10.30 ppm. Signals from protons H3, H5, H6 and H18 to H21 are overlapped between 6.5 and 7.5 ppm but form a characteristic fingerprint profile. For sildenafil, as illustrated in Figure 2B, several typical signals are detected as follows with δ (ppm) and J (Hz): 8.33 d (2.1) (H15), 7.90 dd (8.7, 2.1) (H17), 7.30 d (8.7) (H18), 2.42 s (H29), 1.90 m (H12), 1.51 t (7.1) (H21), and 0.99 t (6.8) (H13). These NMR data corroborate previous findings [15], and both compounds were unambiguously attributed thanks to an in-house database. 

In addition to sugars and adulterants, minor signals of other compounds were identified, as shown in the spectrum of sample **37** (Figure 2C). In 23 out of 50 samples, typical signals of 5-hydroxymethylfurfural (5-HMF) were detected as follows: δ (ppm) 9.52 s, 7.35 d (3.6 Hz), and 6.58 d (3.6 Hz). 5-HMF is an aldehyde-furan compound formed during the thermal decomposition of sugars; it is an indicator of quality deterioration as a result of excessive heating or storage [24]. Other organic acids can be detected, such as acetic acid in 24 samples (singlet, 1.97 ppm) and formic acid in 11 samples (singlet, 8.52 ppm), both known degradation products of sugars [25]. Ethanol, potentially induced by fermentation during storage, was detected in three samples, with only its triplet at 1.17 ppm being discerned, while its corresponding quadruplet was overlapped with signals from sugars. Another fermentation product, 2,3-butanediol [26], was detected in 30 samples from its characteristic doublet at 1.12 ppm d (6.3 Hz). At last, a broad signal at 1.3 ppm, corresponding to CH_2_ of fatty acid chains—known as minor lipid constituents in honey [27]—was observed in 41 samples. Identification of these compounds was achieved by spiking with authentic standards using the benchtop spectrometer, and confirmation was done by HF NMR analysis at 500 MHz. All the data for additional compounds detected are reported in Table S1. 

#### 2.2.2. Insights from PCA and Outlier Spectral Profiles

**Figure 3:**
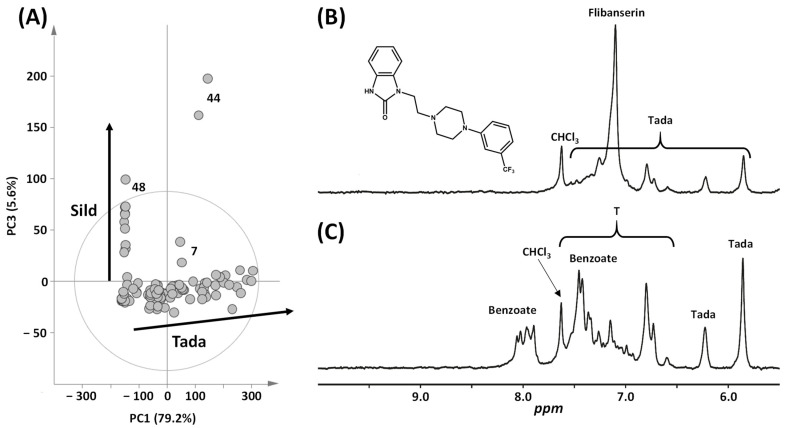
Qualitative analysis of honey samples. (**A**) Score plot of a PCA built from the benchtop ^1^H NMR data of all the samples. Benchtop ^1^H NMR spectra of outlier samples (9.0–5.6 ppm): (**B**) sample **7** and (**C**) sample **44**. Sild: sildenafil, Tada: tadalafil.

First, an unsupervised principal component analysis (PCA) was conducted on all samples, offering an insightful initial overview of the dataset’s structure and revealing clustering trends, as depicted in Figure 3A. The PCA was built from the LF NMR spectra limited to the region 5.6–9.0 ppm of all samples. Other parts of the NMR spectra were excluded. Two cluster-like structures appear to be spread out lengthwise along axes 1 and 3 in the PCA score plot. The first one corresponds to the honey samples containing tadalafil, while the second cluster-like structure comprises samples containing sildenafil. The direction of the arrows indicates increasing concentrations of adulterant, either tadalafil along axis 1 or sildenafil along axis 3. Samples without adulterants are observed at the base of the arrows. Two samples appear as outliers: the first one is number **44** along axis 3, as observed in Figure 3A, and the second one is sample **7**, which is an outlier along axis 2. Sample **48**, containing sildenafil, can be considered an outlier due to its high concentration of sildenafil, as shown later. 

Upon revisiting the NMR spectra of the two outliers, we found that these samples exhibit atypical NMR profiles. In the spectrum of sample **7**, in addition to the presence of tadalafil signals, another active compound has been detected by a broad signal at 7.10 ppm (Figure 3B). Although not all signals are observable, these NMR data are consistent with the aromatic protons of flibanserin. To confirm the identification, we reached HF NMR experiments that allow the detection of characteristic protons as follows: δ (ppm) 7.40 t (1H, H Ar); 7.19–7.03 m (7H, H Ar); 4.00 t (2H, CH_2_-N-(C=O)); 2.72 t (2H CH_2_-N<); 2.68 broad m (4H, CH_2_ piperazine). Furthermore, high-resolution mass spectrometry (HRMS) experiments by direct infusion revealed an MH^+^ ion at *m*/*z* 391.1747, leading to major MS/MS fragments at *m*/*z* 161 and 119. These complementary analyses confirmed the presence of flibanserin [3,28]. This drug was originally developed as an antidepressant medication but was approved by the FDA in 2015 for hypoactive sexual desire disorder in premenopausal women. In the spectrum of sample **44**, two additional multiplets in the aromatic region at 8.2–7.8 ppm and 7.5–7.3 ppm indicate the presence of benzoate (Figure 3C). This was confirmed by spiking the sample with an authentic standard at both 60 and 500 MHz. While this presence could be interpreted as a natural occurrence, the relatively high amount suggests that benzoate was likely added as a preservative to prevent spoilage and halt fermentation in this honey [29]. This is consistent with the absence of 5-HMF in this sample.

### 2.3. Quantitative Analysis

#### 2.3.1. Benchtop NMR Quantitative Analysis 

**Figure 4:**
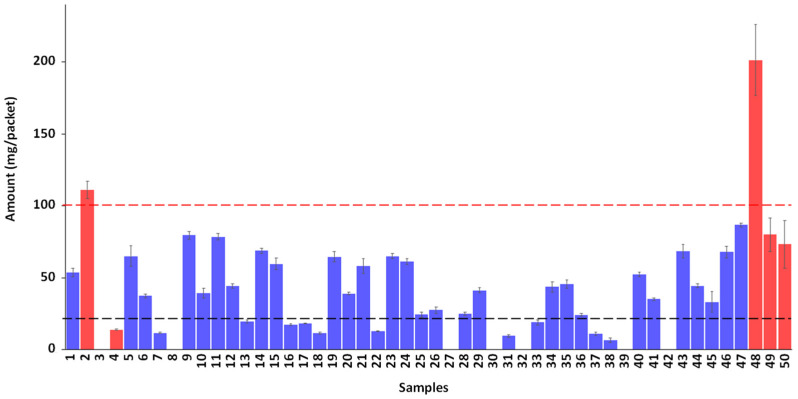
Quantitative analysis of honey samples. Tadalafil quantification is reported in blue ■ and sildenafil in red ■. The dashed lines represent the maximum recommended therapeutic dose once a day for tadalafil (----) in black and sildenafil (----) in red.

NMR quantification can be done by integrating isolated signals of each adulterant. Upon revisiting the spectra of sildenafil and tadalafil in Figure 2, it appears that signals of protons H1 and H8 can easily be integrated for tadalafil, while signals of H15 were chosen for sildenafil. Indeed, the signals of H17 and H18 are close to the CHCl_3_ or 5-HMF signals, respectively, which could hinder integration. Conventionally, quantitative HF NMR (qNMR) experiments are carried out with full relaxation of targeted resonances. However, in benchtop NMR, due to the loss in sensitivity, working in total relaxation conditions requires an increase in the experiment recording time. Indeed, under our experimental conditions, quantifying the adulterant with a minimum signal-to-noise ratio of 10 would necessitate about 1 h of NMR recording. Given that adulterants tadalafil and sildenafil are readily available commercial products, an alternative method of quantification using calibration curves was chosen to reduce recording time by a factor of 4. We already demonstrated in a previous study the relevance of this approach [30]. The areas of each targeted signal of tadalafil and sildenafil were plotted against concentrations in mg/mL. The linearity of the calibration curves was estimated through the calculation of their determination coefficients. The retained concentration for tadalafil was determined as the mean of the values obtained for each proton, taking into account the signals from both H1 and H8. Moreover, this result was validated with an HPLC–UV assay as a standard reference method. The relationship between the concentrations measured by LF NMR and by HPLC is good, as demonstrated by a determination coefficient R^2^ of 0.9771 and a slope of 1.02 between benchtop NMR measurements compared to HPLC. All quantitative results for the assay of tadalafil or sildenafil amount in each honey packet are reported in Table S2 and in the histogram in Figure 4. 

#### 2.3.2. Quality Control Issues 

This study reveals that among the 50 samples, 76% were adulterated with tadalafil and 10% with sildenafil, while 14% were natural or non-adulterated samples. The mere presence of a hidden medicinal substance presents a clear risk to the consumer. Furthermore, as illustrated in Figure 4, most of the tadalafil-adulterated samples (28 out of 38) are above the maximum recommended therapeutic dose of 20 mg. Similarly, although the number of samples adulterated with sildenafil is lower, two out of five exceed the therapeutic dose of 100 mg. The detection of elevated levels of undisclosed active pharmaceutical ingredients in honey-based supplements poses a potential danger to consumers, especially those with chronic illnesses or sensitivities to drug interactions. Such supplements thus present significant life-threatening risks and underscores substantial public health concerns [31,32]. 

Regarding sample **7**, in addition to 12 mg of tadalafil, the presence of about 25 mg of flibanserin raises concerns about a potentially hazardous drug combination. To the best of our knowledge, no toxicological data are available for this combination. The most common adverse events associated with flibanserin are sedation and hypotension [33]. The amount of the preservative sodium benzoate in sample **44** has been assessed at 11 mg by packet, which is well below the acceptable daily intake [34] and poses no additional risk to the consumer, given the presence of tadalafil at 44 mg, which is more concerning.

Our findings highlight the importance of vigilance in monitoring the safety and integrity of food products, particularly those with purported health benefits like honey-based supplements.

### 2.4. Benchtop NMR and Chemometrics: A Comprehensive Screening Approach

#### 2.4.1. Chemometric Workflow

**Figure 5:**
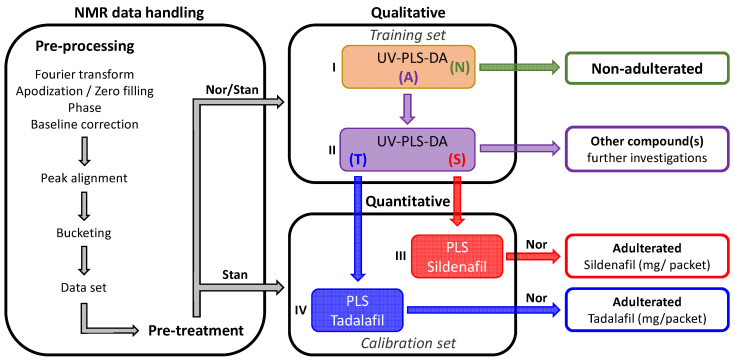
Chemometric workflow for screening of honey samples from benchtop NMR data. NMR data handling includes pre-processing and pre-treatment procedures. The qualitative block enables classification using partial least squares discriminant analysis with unit variance scaling (UV-PLS-DA) on the training set, honey samples as either adulterated (A) or non-adulterated (N), as well as detecting the presence of tadalafil (T) or sildenafil (S). The quantitative block predicts the levels of adulterant, tadalafil or sildenafil, utilizing the PLS regression model on the calibration set. Nor: normalization, Stan: standardization.

After conducting a comprehensive analysis of the samples outlined above, the aim of this section is to emphasize the effectiveness of benchtop NMR coupled with chemometric analysis as a reliable screening tool. We propose a chemometric pipeline for analyzing LF ^1^H NMR spectra as an alternative approach for monitoring the quality of honey supplements (Figure 5).

The objective of this workflow is to offer a quick and straightforward screening of honey samples with minimal knowledge of NMR. This involves addressing the following questions: (i) Is the sample adulterated?; (ii) if adulterated, is it adulterated with sildenafil or tadalafil?; and (iii) what is the predicted content of tadalafil or sildenafil for these adulterated samples?

The proposed workflow (Figure 5) is divided into three blocks. The first one, the NMR data handling, begins after the sample preparations and includes classical pre-processing and pre-treatment of the NMR spectra. Depending on the following blocks, the pre-treatment procedure varies: the samples of the training set were normalized (Nor), while the spectra of the calibration set can be directly used. The key to making this workflow accessible to everyone is to standardize (Stan) the dataset by an internal reference of known and fixed concentration; TSP was used in our experiments (see Section 3.2.2).

**Figure 6:**
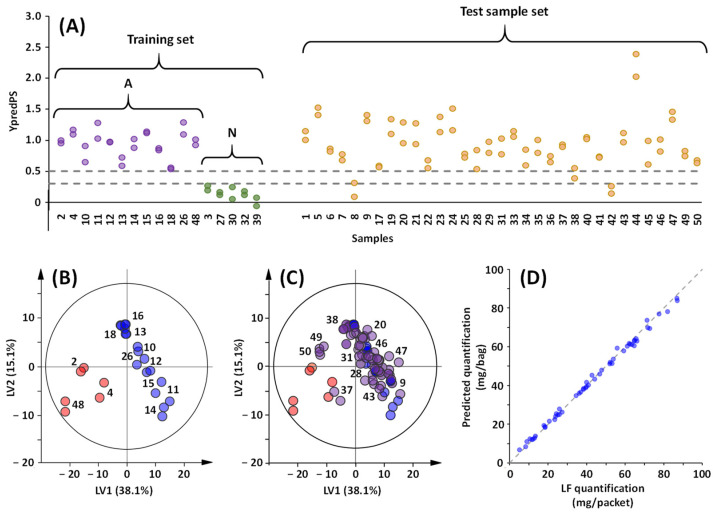
Classification and quantification from benchtop ^1^H NMR spectra of the honey in the test sample set. (**A**) Predicted Y-values (YpredPS) of the test set (orange) based on the two-class UV-PLS-DA model comparing adulterated and non-adulterated samples. The model was generated on the training set with known adulterated (A, purple) and non-adulterated (N, green) samples. The dashed lines represent the limit between the two classes. (**B**) Score plot of the UV-PLS-DA model built on the training set between samples T (adulterated with tadalafil, in blue) and S (adulterated with sildenafil, in red). (**C**) Predicted score plot shows the projection of honey in the test set (in purple) for samples previously classified as adulterated. (**D**) Comparison for tadalafil between LF NMR quantification and predicted levels given by the PLS regression models built on the calibration set.

The second block is the qualitative part of the workflow. The aim is to determine whether a sample of honey is adulterated or non-adulterated, as well as whether it contains tadalafil or sildenafil. For this purpose, partial least squares discriminant analysis (PLS-DA) with unit variance (UV) scaling, i.e., UV-PLS-DA, was used. First, a predicted UV-PLS-DA model (Figure 5, I) between the two classes N and A was obtained with the training set, where samples (T) and (S) were considered together. The predicted Y-values (YpredPS) were generated and used to define class membership [35,36]. The non-adulterated samples (N) have YPredPS in the range of −0.06 to 0.27, while the YPredPS of the adulterated (A) samples is between 0.54 and 1.29 (Figure 6A). The limit value of 0.54 corresponds to sample **18**, with the lowest content of adulterant (11.7 mg/packet of tadalafil) in the training dataset. Based on these values, it is possible to delineate the lowest and the highest limit of the threshold [37]. Within these limits, set at 0.30 and 0.50, test samples will be further considered as borderline. Samples from the test set classified as adulterated and borderline will be selected for the next step. The second UV-PLS-DA (Figure 5, II) was carried out between the two classes (T) and (S); the score plot of this predictive PLS-DA shows a clear discrimination between the two classes of adulterants (Figure 6B). Then, the test sample set is projected into the active PLS-DA, and the predicted score plot is used to identify the adulterant.

The last block is the quantitative aspect of the workflow. The predictive quantification of tadalafil or sildenafil can be reached based on the statistical PLS regression models of solutions of standards with known concentrations (Figure 5, III and IV). PLS models without scaling were built on the calibration set, focusing on signals H1 and H8 for tadalafil and H15 for sildenafil. The weak values (<0.2) of the root-mean-square error of estimation (RMSEE) and the root-mean-square error of cross-validation (RMSECV) confirm the goodness of the fit and the calibration model accuracy [38,39]. The resulting concentrations in mg/mL were then converted to mg/packet (using the normalization step as described in Section 3.5). The concentration of tadalafil or sildenafil in the test sample set can be predicted according to the classification provided by the qualitative block of the workflow.

#### 2.4.2. Blind Validation of the Chemometric Workflow 

A test sample set (*n* = 33) underwent the complete pipeline process, depicted in Figure 5, to validate the proposed workflow. After pre-processing and pre-treatment, the classification of the test samples as adulterated or non-adulterated was performed based on the YPredPS values obtained from the first UV-PLS-DA model. Samples **8** and **42**, with a mean YPredPS value of 0.20 (Table 1), which is lower than the threshold of 0.30, were classified as non-adulterated, as expected. For the remaining samples, YPredPS values ranged from 0.58 to 1.53 (Table 1), leading to their classification as adulterated, except sample **38** (0.47), classified as borderline with a YPredPS value close to the upper threshold limit due to the lowest concentration of the adulterant (6 mg/packet). The elevated YPredPS value of sample **44** (2.20) can be attributed to the presence of benzoate in addition to tadalafil. At the end of this step, without prior information, only samples **8** and **42** were excluded from the next step. 

To proceed further, a second UV-PLS-DA model was carried out to classify the remaining samples (31) as adulterated by either tadalafil or sildenafil. Twenty-seven samples out of thirty-one were located in the (T) group, while three samples were closer to the (S) group (Table 1) on the predicted score plot (Figure 6C). Only sample **44** was deemed to be an outlier, as it fell outside the 95% confidence region of the model based on Hotelling T2, suggesting it could not be adulterated by either tadalafil or sildenafil. This observation aligns with the previously demonstrated presence of benzoate in this sample. The second misclassified sample is sample **37**, which contained tadalafil (11 mg/packet) but was erroneously classified as adulterated by sildenafil. This misclassification can be attributed to the presence of a rather intense doublet of 5-HMF at 7.35 ppm, which is close to the chemical shift of the H18 signal of sildenafil (7.30 ppm) and exhibits the same multiplicity. Hence, in the qualitative part of the proposed workflow, misinterpretation occurred in just 2 samples out of 33 (≈6%), marking a notably successful outcome.

Predictions of content were made, thanks to the PLS regression models on the calibration set, for the resulting 29 samples, 2 containing sildenafil and 27 containing tadalafil, with samples **37** and **44** excluded. The predicted concentration in mg/mL was converted to mg/packet (Table 1) and then compared with the NMR quantification (Figure 6D). The relationship is quite good, with a slope of 0.96, an R^2^ of 0.996 and a mean deviation of 1.6 ± 5.8%. Only two samples have a mean deviation higher than 10% (Table 1), sample **38** (14.3%) and **31** (21.4%), which can easily be explained by a low concentration of tadalafil. The measured and predicted concentrations were, respectively, 7 ± 2 and 8 ± 2 mg/packet for sample **38** and 10 ± 1 and 12 ± 1 mg/packet for sample **31** (Table 1 and Table S2). The proposed approach, which has already proven its effectiveness with other spectroscopic methods [39], is also perfectly suited for analyzing data from benchtop NMR experiments.

## 3. Materials and Methods

### 3.1. Materials

Fifty honey-based supplement samples seized by French customs from 2020 to 2022 were analyzed before the expiry date. Tadalafil and sildenafil citrate were purchased from Acros organics (Geel, Belgium); maltose monohydrate from Alfa Aesar (Haverhill, MA, USA); fructose, D-glucose, sucrose, sodium 2,2,3,3-tetradeutero-3-(trimethylsilyl) propanoate (TSP), 2,3-butanediol, benzoic acid and 5-hydroxymethylfurfural (5-HMF) were obtained from Sigma Aldrich (Saint Quentin Fallavier, France), formic acid and ethanol from Merck (Darmstadt, Germany) and acetic acid from VWR Chemicals (Rosny-sous-Bois, France). Deuterated solvents were obtained from EurisoTop (Saint Aubin, France). 

### 3.2. Sample Preparation

#### 3.2.1. Preliminary Extraction Tests

Optimization of extraction conditions was conducted on samples **1**, **2**, **4**, **5** and **6**. The following solvents were tested: methanol-d4, in the mixture methanol-d4:CDCl_3_ (80:20, *v*/*v*), the mixture methanol-d4:CDCl_3_ (70:30, *v*/*v*) and the mixture methanol-d4:CDCl_3_ (60:40, *v*/*v*). The mixture methanol-d4:CDCl_3_ (60:40, *v*/*v*) yielded a better recovery rate and was chosen as the extraction solvent. For instance, in the extraction of tadalafil from sample **1**, an extraction loss of 20% and 44% is observed with a 70:30 or 80:20 mixture, respectively. Therefore, three successive extractions were tested using 1 g of honey for tadalafil (**1**, **5** and **6**) and sildenafil (**2** and **4**) samples with the selected 60:40 mixture. For the assay of extracted adulterants at each step of the biphasic extractions, a ^1^H quantitative NMR at 500 MHz was carried out on both phases (see Section 3.8). 

#### 3.2.2. Honey Samples

One gram of honey was resuspended in 1 mL of the mixture methanol-d4:CDCl_3_ (60:40, *v*/*v*). The solution was vortexed for 30 s and sonicated for 10 min before being vortexed again for another 30 s. The solution in a closed tube was then centrifuged for 5 min (3000 rpm, 4 °C). A two-phase solution was obtained, with the upper phase containing the compounds of interest in the mixture CDCl_3_:Methanol-d4, while the lower phase contained a mixture of the aqueous sugar honey matrix in methanol-d4. The upper phase was removed with a glass syringe, while the remaining lower phase underwent a second extraction with 1 mL of the same solvent mixture following identical steps. Then, the phases containing the compounds of interest from the two extractions were pooled. At last, for NMR, 600 µL of the final solutions was transferred to an NMR tube, and 30 µL of a 5 mM solution of TSP in methanol-d4 was added as an internal chemical shift and quantification reference. All samples were analyzed in duplicate. For HPLC analysis, 100 or 200 µL of the final solution was evaporated to dryness in a SpeedVac concentrator.

#### 3.2.3. NMR Calibration Samples

Calibration samples were prepared from standard sildenafil and tadalafil in methanol-d4:CDCl_3_ (60:40, *v*/*v*). Ten solutions for tadalafil (0.25, 0.1, 0.5, 0.75, 1, 2, 3, 4, 5, 10 mg/mL) and five solutions for sildenafil (2, 3, 4, 5, 10 mg/mL) were prepared in triplicate. 

### 3.3. HPLC

The HPLC analysis was used as the gold-standard method on the same samples used for NMR. Following extraction and as described above, the dried fraction was dissolved in the mixture CH_3_CN:H_2_O (50:50). To ensure complete solubilization, the sample underwent vortexing for 30 s, followed by sonication for 2 min and then another 30 s of vortexing before filtration using PTFE filters (0.45 µm) prior to injection.

The HPLC system was an Agilent 1260 Infinity II model with a diode array detector. The analysis conditions were reversed-phase column C18 Waters Sunfire (100 × 4.3 mm; 3.5 µm particle size) maintained at 30 °C; mobile phase (A) UHQ water and (B) CH_3_CN (analytic grade for LC), both containing 0.05% trifluoroacetic acid (*v*/*v*); flow rate, 1.0 mL/min; volume of injection, 5 µL; detection, UV detection at 284 nm. The elution profile was as follows: 0–1 min, isocratic elution with A:B mixture in 20:80 ratio; 1–12 min, linear gradient from 20:80 to 40:60 A:B ratio; 12–14 min, linear gradient from 40:60 to 10:90 A:B ratio; 14–16 min column washing out with 10:90 A:B mixture; 16–19 min, return to 20:80 A:B mixture ratio; and, finally, the system was left to stabilize for 6 min between consecutive injections. For quantification of honey samples, calibration curves were obtained with solutions between 0.02 mg/mL and 0.2 mg/mL of tadalafil (R^2^ = 0.9991) and between 0.05 and 1 mg/mL for sildenafil (R^2^ = 0.9997).

### 3.4. Low-Field NMR Analysis

Spectra were acquired on a Pulsar^TM^ benchtop NMR spectrometer (Oxford Instruments, Abingdon, UK) operating at a frequency of 59.7 MHz for ^1^H. The temperature inside the spectrometer was 310 K. The acquisition was performed with SpinFlow 2.3.0 software (Oxford Instruments) and the processing with MNova 14.0 (Mestrelab Research, Santiago de Compostela, Spain).

A DANTE sequence was performed in order to suppress the water signal. Free induction decays (FIDs) were recorded with a flip angle of 90° (11.3 µs), a spectral width of 5000 Hz, and 8 K complex points (acquisition time of 1.64 s). The relaxation delay was set at 2 s, and 256 transients were recorded, leading to a total acquisition time of 18 min. For the DANTE scheme, parameters were optimized and fixed as follows: δ delay at 300 µs, low amplitude pulse p1 at 1 µs, number of loops at 1000 (n × m). The total time was 600 ms for a frequency width of the excitation at 36 Hz.

For data processing, the FIDs (free induction decays) were apodized with an exponential filter (line broadening (LB) of 0.3 Hz), and a manual baseline correction was performed. The number of points was increased to 16 K in Fourier-transformed spectra. The signal of TSP set at 0 ppm was used as an internal reference for chemical shift (δ) measurement.

T1 relaxation times of tadalafil and sildenafil in honey sample solutions were measured at 2.3 s and 1.2 s for H8 and H1 of tadalafil, respectively, and 1.9 s for H15 of sildenafil. The TSP protons had a longer relaxation time of 3.1 s. T1s were measured by the classical inversion-recovery pulse sequence method. 

### 3.5. Benchtop NMR Quantitative Analysis

For NMR quantification, calibration curves for tadalafil were obtained from a mean of 4 curves, i.e., 40 solutions, and have the following characteristics: R^2^ = 0.9977, y = 3158x + 153 (H1) and R^2^ = 0.9963, y = 1302x + 80 (H8) while for sildenafil, data are obtained from a mean of 3 curves, i.e., 15 solutions: R^2^ = 0.9981, y = 1137x + 38 (H15). The quantification of adulterants in honey samples was obtained by direct comparison of the area of H1 and H8 for tadalafil and H15 for the sildenafil signal with the data from the calibration curve. The amount of compound in mg by packet was calculated from the following equation:

$$Q=C_{A}\times V\times \frac{m_{packet}}{m}$$

with *C_A_* the concentration of adulterant (S or T) in the solution analyzed, *V* the volume withdrawn of the sample solution, *m_packet_* the weight of honey per packet and m the mass of the honey used for the NMR assay. 

### 3.6. Dataset

Representative commercial honey samples were used for the training set, comprising 5 non-adulterated samples (designated as N, samples **3**, **27**, **30**, **32**, and **39**), 9 samples adulterated with tadalafil (designated as T, samples **10**–**16**, **18** and **26**) with concentrations ranging from ≈12 to 79 mg/packet, and 3 samples adulterated with sildenafil (designated as S, samples **2**, **4** and **48**) at concentrations of 111, 14 and 201 mg/packet, respectively. Calibration samples at different known concentrations were used for this dataset, consisting of 40 spectra for tadalafil and 15 spectra for sildenafil. Spectra and their known concentrations correspond to the set of independent and dependent variables, respectively. The test sample set comprises 33 honey samples, including 2 samples without adulteration (samples **8** and **42**), 2 samples adulterated with sildenafil (samples **49** and **50**), and 29 samples adulterated with tadalafil (samples **1**, **5**–**7**, **9**, **17**, **19**–**25**, **28**, **29**, **31**, **33**–**38**, **40**, **41**, **43**–**47**), for a total of 66 spectra.

### 3.7. Chemometrics 

#### 3.7.1. Data Handling

All multivariate statistical analyses were conducted using SIMCA-P+ 13.0.3.0 software (Umetrics, Umea, Sweden). Pre-processing involved aligning spectra and performing automatic binning (0.01 ppm) using the Align Spectra and Chemometrics module in MNova 14.0.1 software, following the classical NMR data processing as described in Section 3.4. Only the fingerprinting region 5.6–9.0 ppm of the benchtop spectra, excluding the residual solvent signal of chloroform (7.55–7.75 ppm), was used for both solutions of standards and honey supplement samples. 

For data pre-treatment, all datasets were standardized by dividing their areas by that of the internal standard TSP. According to the workflow proposed in this article, the commercial samples dataset could be normalized before or after statistical analysis using the equation described in the Section 3.5, where C_A_ is replaced by the value obtained after the pre-processing procedure. 

Dataset structure exploration involved generating principal component analysis (PCA) with mean-centered scaling on all spectra of the honey samples (100 spectra) to find trends and detect outlier samples.

#### 3.7.2. Qualitative Statistical Analysis

Partial least squares discriminant analysis (PLS-DA) was initially performed on the training set with qualitative variables (N) and (A) = (T) + (S), comprising 34 spectra, followed by a second one with the two qualitative variables (T) and (S), consisting of 24 spectra. Unit Variance (UV) scaling was chosen to compress the amplitude variations of variables in the dataset. The UV-PLS-DA model led to a predictive model in both cases with well-validated criteria: 3 components with R^2^X = 0.58; 0.61, R^2^Y = 0.81; 0.97, Q^2^ = 0.54; 0.81 and CV-ANOVA = 7.7 × 10^−4^; 4.4 × 10^−6^. Sample classification was done according to the chemometric workflow using the predicted Y-values (YPredPS) or the predicted score plot. A YPredPS value close to 0 or 1 indicates that the sample belongs to the adulterated or the non-adulterated class, respectively. The defined thresholds for Y-values are as follows: YPredPS < 0.30 indicates that the honey sample is not adulterated; for 0.30 < YPredPS < 0.50, the honey sample is considered borderline and may have a low content of adulterant; and YPredPS > 0.50 indicates that the honey sample is adulterated.

#### 3.7.3. Quantitative Statistical Analysis

The prediction of the content of tadalafil or sildenafil was achieved thanks to the PLS regression models generated on the calibration dataset, based on the specific NMR signals of tadalafil H1 and H8 (5.6–6.4 ppm) and sildenafil H15 (8.10–8.50 ppm). The performance of the PLS model was evaluated and validated with the following criteria: R^2^Y > 0.999 and Q^2^ > 0.998. The optimal number of PLS components was selected based on the lowest value of the root-mean-square error of cross-validation (RMSECV), 0.17 (2 components) and 0.11 (3 components) for tadalafil and sildenafil, respectively. The PLS model was also evaluated by the root-mean-square error of estimation (RMSEE) on the calibration set and the root-mean-square error of prediction (RMSEP) obtained on the test set. The RMSEE was 0.14 and 0.09 for tadalafil and sildenafil, respectively. The value of RMSEP was closer than RMSECV for tadalafil, confirming the robustness of the model. RMSEP was not used for sildenafil due to the low number of samples in the test set. Once the predictive quantification was obtained, samples were normalized as previously described.

### 3.8. High-Field NMR Analysis

HF ^1^H NMR experiments were performed on a Bruker Avance 500 spectrometer at 298 K. Typical acquisition parameters were as follows: number of scans 64, 30° pulse, acquisition time 5.45 s, spectral width 6000 Hz, 64 K data points, and relaxation delay 9.55 s. Data processing was performed with Topspin software 3.2. The concentrations were measured by comparing the signal areas of convenient protons of targeted compounds with that of TSP.

### 3.9. Mass Spectrometry

Samples were dissolved in methanol and analyzed after direct infusion in a Waters XEVO G2 QTOF mass spectrometer in positive electrospray ionization mode (ESI+). Instrument parameters were set as follows: for MS analysis, cone voltage 15 V and 30 V, scan range *m*/*z* 100–1200; for MS/MS analysis, three different collision energies of 15, 25 and 35 eV were used, with cone voltage maintained at 30 V and scan range *m*/*z* 50–1200.

## 4. Conclusions

In the field of benchtop NMR spectroscopy, future developments are expected to focus on enhanced automation, encompassing sample preparation, online benchtop NMR analysis, and data treatment. This study makes a valuable contribution to the data treatment step by introducing an original workflow accessible to non-NMR specialists. The proposed workflow enables blind analysis of test samples once a model is validated, facilitating not only sample classification but also quantification of adulterants. It is important to remember that hidden active ingredients may pose risks for consumers, especially at higher doses. This approach holds promise for streamlining quality control processes and enhancing the efficiency of adulteration detection in regulatory agencies or customs laboratories.

## Figures and Tables

**Table 1:** Classification and levels of adulterated samples in the test sample set (*n* = 33), thanks to the chemometric workflow. The classification was performed by the predicted YPredPS value based on the UV-PLS-DA I model, (A) vs. (N), followed by the visual observation of the projection of the samples on the UV-PLS-DA II model, the (T) vs. (S) model shown in Figure 6B,C. Predicted concentrations of tadalafil and sildenafil were obtained by their corresponding PLS regression models III and IV.

	Qualitative				Quantitative	
	Predictive YpredPS UV-PLS-DA I		Projection UV-PLS-DA II		Predicted Content PLS III (S) or IV (T)	Predicted PLS versus Benchtop NMR
N°	YPredPS	Class	Class	Adulterant *	Levels (mg/Packet)	SD
1	1.08	A	T	Tadalafil	56 ± 6	3.5
5	1.47	A	T	Tadalafil	66 ± 12	1.0
6	0.84	A	T	Tadalafil	37 ± 1	−0.3
7	0.73	A	T	Tadalafil	12 ± 1	4.0
8	0.20	N	-	-	-	-
9	1.36	A	1	Tadalafil	78 ± 1	−2.5
17	0.58	A	T	Tadalafil	19 ± 1	2.0
19	1.22	A	T	Tadalafil	62 ± 2	−3.9
20	1.12	A	T	Tadalafil	39 ± 1	0.0
21	1.11	A	T	Tadalafil	58 ± 6	−0.9
22	0.61	A	T	Tadalafil	13 ± 1	3.1
23	1.25	A	T	Tadalafil	64 ± 1	−2.0
24	1.53	A	T	Tadalafil	61 ± 1	−0.5
25	0.75	A	T	Tadalafil	24 ± 2	−2.9
28	0.69	A	T	Tadalafil	27 ± 2	7.1
29	0.89	A	T	Tadalafil	44 ± 3	5.6
31	0.90	A	T	Tadalafil	12 ± 1	21.4
33	1.10	A	T	Tadalafil	21 ± 2	7.8
34	0.72	A	T	Tadalafil	43 ± 4	−1.1
35	0.90	A	T	Tadalafil	46 ± 4	−0.2
36	0.69	A	T	Tadalafil	24 ± 1	0.8
37	0.91	A	S	Tadalafil	-	-
38	0.47	Borderline	T	Tadalafil	8 ± 2	14.3
40	1.03	A	T	Tadalafil	51 ± 1	−2.3
41	0.73	A	T	Tadalafil	35 ± 1	0.8
42	0.20	N	-	-	-	-
43	1.04	A	T	Tadalafil	65 ± 7	−5.1
44	2.21	A	Outlier	Tadalafil	-	-
45	0.80	A	T	Tadalafil	33 ± 9	−2.1
46	0.92	A	T	Tadalafil	66 ± 5	−2.2
47	1.40	A	T	Tadalafil	84 ± 1	−2.6
49	0.78	A	S	Sildenafil	78 ± 11	−2.1
50	0.65	A	S	Sildenafil	67 ± 12	−9.3

* Identification of adulterants was obtained by conducting NMR characterization of the samples.

## Data Availability

Data are contained within the article and Supplementary Materials.

## Supplementary Materials

The following supporting information can be downloaded at: https://www.mdpi.com/article/10.3390/molecules29092086/s1, Table S1: Naturally occurring compounds detected in honey-based supplements with benchtop NMR.; Table S2: Honey supplements analyzed in this study along with adulterants detected and their benchtop NMR quantification.
