# Supplementary material for: Benchtop NMR Coupled with Chemometrics: A Workflow for Unveiling Hidden Drug Ingredients in Honey-Based Supplements
Source: Molecules. 2024 May 1;29(9):2086. doi: 10.3390/molecules29092086 (PMC11085444; doi:10.3390/molecules29092086)
Supplement: Supplementary file 1 [file molecules-29-02086-s001.zip › Supplementary Tables S1.pdf]

Table S1. Natural occurring compounds detected in the honey-based supplements with benchtop NMR. The table does not include adulterants (see Table S2) or sugars.

**Honey Samples**	5-HMF	acetate	formate	ethanol	2,3-butanediol	fatty acids
1	●	●			●	●
2						
3					●	
4	●					
5		●				
6	●	●			●	●
7		●		●		
8					●	●
9		●				
10	●	●			●	●
11					●	●
12	●	●			●	●
13					●	●
14		●				●
15		●		●		●
16		●	●		●	●
17	●		●			●
18					●	●
19	●	●	●		●	●
20	●	●	●	●		●
21	●	●	●		●	●
22	●		●			●
23	●	●	●		●	●
24		●			●	●
25	●		●		●	
26	●		●			●
27	●		●			●
28	●				●	●
29		●				●
30					●	●
31		●	●		●	●
32					●	●
33		●			●	●
34	●	●			●	●
35	●	●			●	●
36						●
37	●				●	
38						●
39	●				●	●
40	●	●			●	●
41		●			●	●
42	●					●
43	●	●			●	●
44					●	●
45	●				●	●

46					●
47	●			●	●
48					
49					●
50					●
