# Supplementary material for: Benchtop NMR Coupled with Chemometrics: A Workflow for Unveiling Hidden Drug Ingredients in Honey-Based Supplements
Source: Molecules. 2024 May 1;29(9):2086. doi: 10.3390/molecules29092086 (PMC11085444; doi:10.3390/molecules29092086)
Supplement: Supplementary file 1 [file molecules-29-02086-s001.zip › Supplementary Tables S2.pdf]

Table S2. Honey supplements analyzed in this study along with adulterants detected and their benchtop NMR quantification.

Honey Samples	Honey packet content	Adulterant	Benchtop NMR quantification (mg/packet)
1	20 g	Tadalafil	54 ± 3
2	15 g	Sildenafil	111 ± 6
3	10 g	-	-
4	10 g	Sildenafil	14 ± 1
5	30 g	Tadalafil	65 ± 8
6	10 g	Tadalafil	37 ± 2
7	10 g	Tadalafil<br>Flibanserin	12 ± 1<br>23 ± 6 [1]
8	20 g	-	-
9	20 g	Tadalafil	80 ± 3
10	10 g	Tadalafil	39 ± 4
11	15 g	Tadalafil	79 ± 3
12	10 g	Tadalafil	44 ± 2
13	10 g	Tadalafil	20 ± 2
14	30 g	Tadalafil	69 ± 2
15	10 g	Tadalafil	60 ± 4
16	10 g	Tadalafil	17 ± 1
17	10 g	Tadalafil	18 ± 1
18	10 g	Tadalafil	12 ± 1
19	10 g	Tadalafil	65 ± 4
20	20 g	Tadalafil	39 ± 1
21	12 g	Tadalafil	58 ± 6
22	10 g	Tadalafil	13 ± 1
23	20 g	Tadalafil	65 ± 2
24	30 g	Tadalafil	61 ± 2
25	10 g	Tadalafil	24 ± 2
26	20 g	Tadalafil	28 ± 3
27	10 g	-	-
28	20 g	Tadalafil	25 ± 2
29	30 g	Tadalafil	41 ± 2
30	20 g	-	-
31	20 g	Tadalafil	10 ± 1
32	20 g	-	-
33	15 g	Tadalafil	19 ± 2
34	15 g	Tadalafil	44 ± 4
35	10 g	Tadalafil	46 ± 3
36	10 g	Tadalafil	24 ± 1
37	20 g	Tadalafil	11 ± 2
38	20 g	Tadalafil	7 ± 2
39	20 g	-	-
40	10 g	Tadalafil	53 ± 2
41	20 g	Tadalafil	35 ± 2
42	10 g	-	-
43	10 g	Tadalafil	69 ± 5
44	10 g	Tadalafil	44 ± 2

45	15 g	Tadalafil	33 ± 8
46	15 g	Tadalafil	68 ± 5
47	20 g	Tadalafil	87 ± 2
48	20 g	Sildenafil	201 ± 25
49	12 g	Sildenafil	80 ± 12
50	43 g[2]	Sildenafil	73 ± 17 [2]

[1] Quantification of flibanserin was done at 500 MHz with a classical qNMR experiment

[2] This sample was in the form of a viscous paste in a vial. Amount was calculated for 10 g of product.
